# Keeping social distance in a classroom while interacting via a telepresence robot: a pilot study

**DOI:** 10.3389/fnbot.2024.1339000

**Published:** 2024-02-14

**Authors:** Kristel Marmor, Janika Leoste, Mati Heidmets, Katrin Kangur, Martin Rebane, Jaanus Pöial, Tiina Kasuk

**Affiliations:** ^1^IT College, Tallinn University of Technology, Tallinn, Estonia; ^2^School of Educational Sciences, Tallinn University, Tallinn, Estonia

**Keywords:** social presence, telepresence robots, remote communication, proximity, human-robot interaction, social norms, behavioral realism

## Abstract

**Introduction:**

The use of various telecommunication tools has grown significantly. However, many of these tools (e.g., computer-based teleconferencing) are problematic in relaying non-verbal human communication. Telepresence robots (TPRs) are seen as telecommunication tools that can support non-verbal communication.

**Methods:**

In this paper, we examine the usability of TPRs, and communication distance related behavioral realism in communication situations between physically present persons and a TPR-mediated person. Twenty-four participants, who played out 36 communication situations with TPRs, were observed and interviewed.

**Results:**

The results indicate that TPR-mediated people, especially women, choose shorter than normal communication distances. The type of the robot did not influence the choice of communication distance. The participants perceived the use of TPRs positively as a feasible telecommunication method.

**Discussion:**

When introducing TPRs, situations with greater intrapersonal distances require more practice compared to scenarios where a physically present person communicates with a telepresent individual in the audience. In the latter situation, the robot-mediated person could be perceived as “behaviorally realistic” much faster than in vice versa communication situations.

## 1 Introduction

The COVID-19 era pushed various sectors (including business and education) to make wide-scale use of digital technologies that allow people to communicate over distance. These digital technologies provide people with means for communicating, usually allowing them to relay their ideas, visual and voice, while being able to perceive their communication partners' ideas, visual and voice. Compared to face-to-face communication, human presence (i.e., the extent of perceiving the communication parties as real human beings) in digitally relayed conversations is limited. For example, there is no perceivable human body, the role of body language is limited or even absent; people can only be seen partially or they are represented on computer screens only by their names; and their relayed voices could have distortions. These limitations have encouraged academic discussion about maintaining the richness of human communication, inherent to real-world communication, in situations where communication is upheld by digital technologies (Gunawardena, 1995; Lowenthal and Dunlap, 2018; Carillo and Flores, 2020).

In face-to-face communication, information is moving via two different channels: verbal communication and non-verbal communication. When communication is mediated via digital technologies (e.g., teleconferencing systems), the non-verbal side of communication mostly suffers, losing the participants' mimicry, their mutual location, etc. Some digital technologies are better prepared for mediating non-verbal communication. Telepresence robotics is a promising emerging technology that provides people with robotic bodies with limited support to non-verbal communication. In this paper, we are examining an important aspect of non-verbal communication—the intrapersonal distance used when telepresent persons (TPs) communicate with physically present persons (PPs), using a telepresence robot (TPR) as a mediating tool. We lean on the concept of behavioral realism (Freeman et al., 2000), wherein it is proposed that the perceived level of authenticity in a communication scenario, encompassing both verbal and non-verbal elements, plays a role in enhancing the productivity of interaction and the subjective pleasantness experienced by the communicative participants. In our theoretical reasoning, we also make use of papers from earlier periods, as the current research on TPRs is scant (Leoste et al., 2022). In addition, we compare our results with the ones of our previous study (Leoste et al., 2023), introduced in Section 1.3.

### 1.1 TPR-mediated telepresence

**Figure 1:**
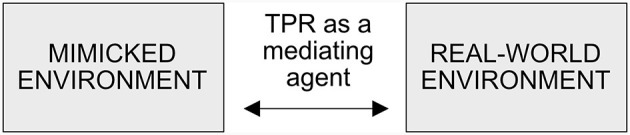
TPR as an agent, mediating the TP's presence between the real-world and mimicked environments.

In 1980, Marvin Minsky, a co-founder of the Artificial Intelligence Laboratory at the MIT and a pioneer in this field, introduced the term “telepresence” to refer to teleoperation systems used for manipulating remote physical objects (Rae et al., 2015). The meaning of the term “telepresence” leans on the concept of “presence” (or its various aspects, such as physical presence or social presence—see Biocca et al., 2003) that characterize the feeling of being present somewhere, felt by a person whose biological body is somewhere else (Freeman et al., 2000). Thus, **telepresence** refers to the sensation, felt by a TP, of being present in a location distant from one's physical location when using a TPR. In other words, telepresence via a TPR allows a real person to experience a real-life environment remotely (El-Gayar et al., 2008), using the mediated sensory input that is provided by a TPR. From the TP's point of view, the TPR is used to mediate a real-life environment in order to create an interactive environment in a remote location (Figure 1). This interactive environment mimics closely the mediated real-world environment, where the TPR is located. In this simulated environment, information and communication technologies (ICTs) provide the TP with realistic sensory input that allows the TP to project their self-image around the TPR (see also Nakanishi et al., 2017) and accept the mimicked environment as real. And, *vice versa*, the TPR as a mediating agent allows the on-site persons to perceive the TP as being present (Figure 1).

The feeling of the TP's presence, as felt by both the PP and TP, depends on several factors, one of them being the TPR's abilities. In case of a typical modern TPR, it is usually a movable, remote-controlled device, equipped with cameras, speakers, microphones, screens, sensor-assisted motion control, and other interactive features designed for remote communication and collaboration. Such a TPR generally supports two-way audio and video communication with persons in remote locations (telepresent persons). Advanced TPRs have features such as laser pointers, auto-navigation, mapping features, and real-time full-resolution zoom that provides UHD 4K resolution of objects like whiteboards (Davey, 2021). However, the ability to manipulate objects in remote environments and receive tactile feedback is still limited.

Controlling a TPR and providing the TP with a mimicked environment require wireless internet connection and a computer or a smart device (Takeuchi et al., 2020; Kaelin et al., 2021), where the mimicked environment is created with the help of the computer's screen and speakers. At the same time, the camera and microphone of the TP's computer are used to project the TP's face and audio onto the TPR's screen and speakers, allowing the on-site persons to communicate with the TP face-to-face, supporting the TP's presence in the real-world environment (Perez, 2020).

Assessing the level of presence subjectively, as felt by the TP, is somewhat difficult as: (a) the feeling is constructed in the awareness of a person as a dynamic sum of sensory information and relevant memories (Freeman et al., 1999); (b) the vocabulary for expressing the degrees of presence is limited (Freeman et al., 2000); and (c) in the situation where presence is measured, the test subject is most likely aware of the true location of their biological body. In order to bypass these limitations, **behavioral realism** can be used as another way of assessing the level of the TP's presence. The behavioral realism approach was originally used to characterize the behavior of simulated beings in simulated environment as perceived by a human that is present in this simulated environment (Guadagno et al., 2007). As TPRs mediate the real-world environments to TPs as mimicked (digitally mediated, limited environments), behavioral realism can also be used to study the behavior of test subjects in these mimicked environments, and compare it with their behavior in real-world environments. Research (Garau, 2003; Bailenson et al., 2005) suggests that lower levels of behavioral realism indicate communication parties' lower perception of social presence, while higher levels of behavioral realism are associated with lesser association of being in a virtual environment.

### 1.2 Interpersonal communication distance

An important component of non-verbal communication is the spatial arrangement between communication partners—how far apart they are and what distance they choose. Over half a century ago, American anthropologist Edward Hall proposed a classification of communication distances (Hall, 1966). According to Hall, people use four different distances in everyday communication, which he called intimate, personal, social, and public distances. Many subsequent studies have confirmed Hall's approach while also pointing out significant cultural differences in people's spatial behavior.

According to Hall, the choice of distance depends on the relationship between communication partners (the closer the relationship, the smaller the distance), as well as the person's emotional state and what they are currently doing (Hall, 1966). It is important to note that the “selection” of distance is not a conscious activity—we do not think or calculate how close or far we should be from other people. It simply happens spontaneously, reflecting the norm that has been established in a society and internalized by people—an appropriate distance and arrangement are subjectively pleasant. Likewise, a violation of distance norms can be unpleasant, for example forced communication at a very close range. The distances described by Hall are as follows:

1. **Intimate distance** is < 50 cm and is used for communication between very closely related individuals, such as a parent and child or spouses. It involves visual, haptic, and olfactory cues.
2. **Personal distance** is ~50–120 cm away and it is used while there is small talk between friends or well-known partners. In this distance, the visual cues are still observed, but haptic and olfactory cues are less significant.
3. **Social distance** is around 120–350 cm away and is used for more formal communication situations, such as talking with strangers or placing customer seats at offices. Personal details are hidden, and communication becomes optional.
4. **Public distance** is more than 350 cm away and is used in public situations such as speeches, lectures or when communicating with superiors. Visual cues are less intimate, and communication relies more on speech volume and gestures.

While Hall's (1966) proximity zones have been supported by subsequent studies, it is important to acknowledge that the size of these zones can be influenced by a range of factors, including cultural background, gender, and other individual characteristics (Lewis, 1998). For instance, Kilbury et al. (1996) discovered that less space was given to disabled individuals than to non-disabled individuals, and that men tended to use longer distances than women did during conversations. Such factors may lead communication parties to prefer different proximity zones, resulting in a situation where the actual communication distance is perceived as inappropriate by one of the parties. Inaccurate proximity distance selection may lead to less desirable communication outcomes due to expectancy violation, where one of the communication parties perceives unexpected social norm violations (Burgoon and Hale, 2009). In addition, in certain situations, such as in education, closer distances can enhance instructional efficiency (Miller, 1978)—or refer to communication party's inability to view robotic bodies as social entities (Walters et al., 2005).

The choice of communication distance in situations, where one of the parties is present via a TPR, is little researched. People's spatial preferences when interacting with a (telepresence) robot can be affected by their gender, pre-existing attitudes toward robots, their experience with robots, their cultural background, robots' appearance, and the interaction's context. For example, Takayama and Pantofaru (2009) found that users' gender may influence perceptions of proximity in human-robot interaction, and Mumm and Mutlu (2011) found that participants who disliked the robot would maintain a greater distance from it, with male participants distancing themselves further than female participants would Joosse (2017) also found significant gender differences in people's spatial behavior. Nonetheless, as suggested by Leichtmann et al. (2021), the current research results in this area are not conclusive.

### 1.3 Research questions

This paper presents the second study of a research cycle focused on behavioral aspects of communication situations between TPs and PPs.

- Study 1: In the first paper by Leoste et al. (2023), we measured the distance chosen by PPs when interacting with TPs. In that study, we focused on an aspect of spatial behavior—interpersonal distance—by examining four social zones: intimate, personal, social, and public. Employing the Double 3 TPRs in simulated situations, we compared interactions with TPRs to in-person interactions. Our findings indicated that, irrespective of status or prior relationships, participants maintained a communication distance similar to normal human-to-human interactions, with participant gender and their experience in computer gaming influencing preferences. This research implied that, in general, TPR-mediated communication adheres to established social norms and may not necessitate additional physical space in educational settings, presenting implications for the integration of TPRs in education.
- Study 2: In this paper, we analyze in greater depth the spatial behavior of TPs when interacting with PPs.

Specifically, we are interested in explaining: (a) to what extent the communication distance selected by a TP, when communicating with a PP, differs from the distance selected by a PP when communicating with a TP; (b) how much the distance chosen by a TP is “behaviorally realistic,” i.e., how it corresponds to the norms of spatial behavior established between physical individuals; and (c) the assessments of individuals communicating in a “TP and PP” situation, regarding the technical suitability and subjective pleasantness of such a communication method. In order to meet these research aims, we have formed the following research questions:

1. What is the communication distance chosen by a TP when communicating with a PP, and does it differ from the communication distance chosen by a PP when communicating with a TP?
2. Does the communication distance chosen by a TP depend on the TP's gender or the type of TPR used?
3. To what extent does the communication distance chosen by a TP differ from the “behaviorally realistic” distance that people maintain when communicating without a robot?
4. How do TPs and PPs evaluate the technical suitability and subjective comfort of communication TPR-mediated communication?
5. Do the assessments of TPs and PPs regarding the technical suitability and subjective comfort of the communication process depend on the type of robot used?

## 2 Method

### 2.1 Telepresence robots

We used three different TPRs in the study: (a) Double 3 by Double Robotics (https://www.doublerobotics.com/tech-specs.html); (b) Ohmni Gen 12 by OhmniLabs (https://ohmnilabs.com/products/ohmni-telepresence-robot/); and (c) TEMI 2 by TEMI (https://www.robotemi.com/product/temi/). All of these robots feature a strongly simplified humanoid shape, having upright posture and a separate head unit attached to the body that is able to move around using a wheeled base at its lower end. TEMI 2 is the shortest with the height of 100 cm. The height of Double 3 is adjustable from 119 to 152 cm, and Ohmni has a fixed height of 146 cm. The head unit contains a tablet-like display for relaying telepresent person's face, a microphone array, speakers and various cameras. While the robots have some limited autonomous movement abilities, normally their moves are controlled by a TP via a browser-based interface. None of these robots has movable arms or other manipulators for opening doors, holding objects or for other similar activities.

### 2.2 Sample

The sample for the study (*N* = 24) consisted of teaching staff and administrative employees from different Estonian and foreign universities. The people in the sample had varying experiences with telepresence robots: some had not encountered them at all, some had encountered them briefly, and some had briefly used them in their work. The sample included both men (*N* = 9) and women (*N* = 15), as well as people from different nationalities. Half of the sample (*N* = 12, of these three male and nine female) took the role of TP; the other half (*N* = 12) were PPs. The roles did not change during the experiments. Next, the sample was divided into four groups of six members each (three TPs and three PPs). The groups were formed to be as heterogeneous as possible: equal representation of different genders, different nationalities, different institutions, and different levels of experience. Each participant was given a unique code to use in data collection. In experiments, each TP used TPRs three times (each time a different TPR, 36 iterations in total). In average, participants of both genders used TPRs equally to communicate with PPs of both gender equally. After excluding the iterations with invalid measurements, 33 valid iterations remained.

### 2.3 Ethics

Written consent was obtained from all participants before the experiment. The participants signed a consent form, stating that personalized data would not be collected. The form also stated that all photos would be blurred for research purposes, and videos would be deleted after analyzing observation data. Studies involving human subjects: Ethical review and approval was not required for the study on human participants in accordance with the local legislation and institutional requirements.

### 2.4 Experiment

In our previous study (Leoste et al., 2023) we had identified three general communication scenarios that happen when in-person and telepresent persons interact: (a) ordinary verbal discussion between two parties; (b) the in-person party demonstrates an object (a page with text and figures) to the telepresent party; and (c) the telepresent party demonstrates an object (a page with text and figures) to the in-person party. These observations were used to create scenarios for communication situations in this study. The scenarios made use of a common topic—choosing a restaurant, as it allows all participants to contribute on relatively equal bases.

The experimental situation in this study is a 3-part short-term communication situation between a person and a person mediated via a TPR. During the situation, the TPR-mediated person must first approach the PP, chat with them, next share information on the robot's screen, and then read information from a piece of paper in the hands of the PP. All these parts followed the general scenario of choosing a restaurant, while the information shared was a menu of a restaurant.

#### 2.4.1 Room setup and process

Three types of rooms (five rooms in total) were used to conduct the experiment: (a) a typical university study room as the communication room where the communication situation was played out; (b) three smaller rooms were used as the control rooms where TPs controlled TPRs; and (c) a typical university study room were workshops were held for waiting participants (the workshops were not related to robotics or telepresence). The robots and the computers that were used to control them were connected to a separate Wi-Fi internet router that operated on a 5 GHz frequency with a speed of at least 10 Mbit/s.

**Figure 2:**
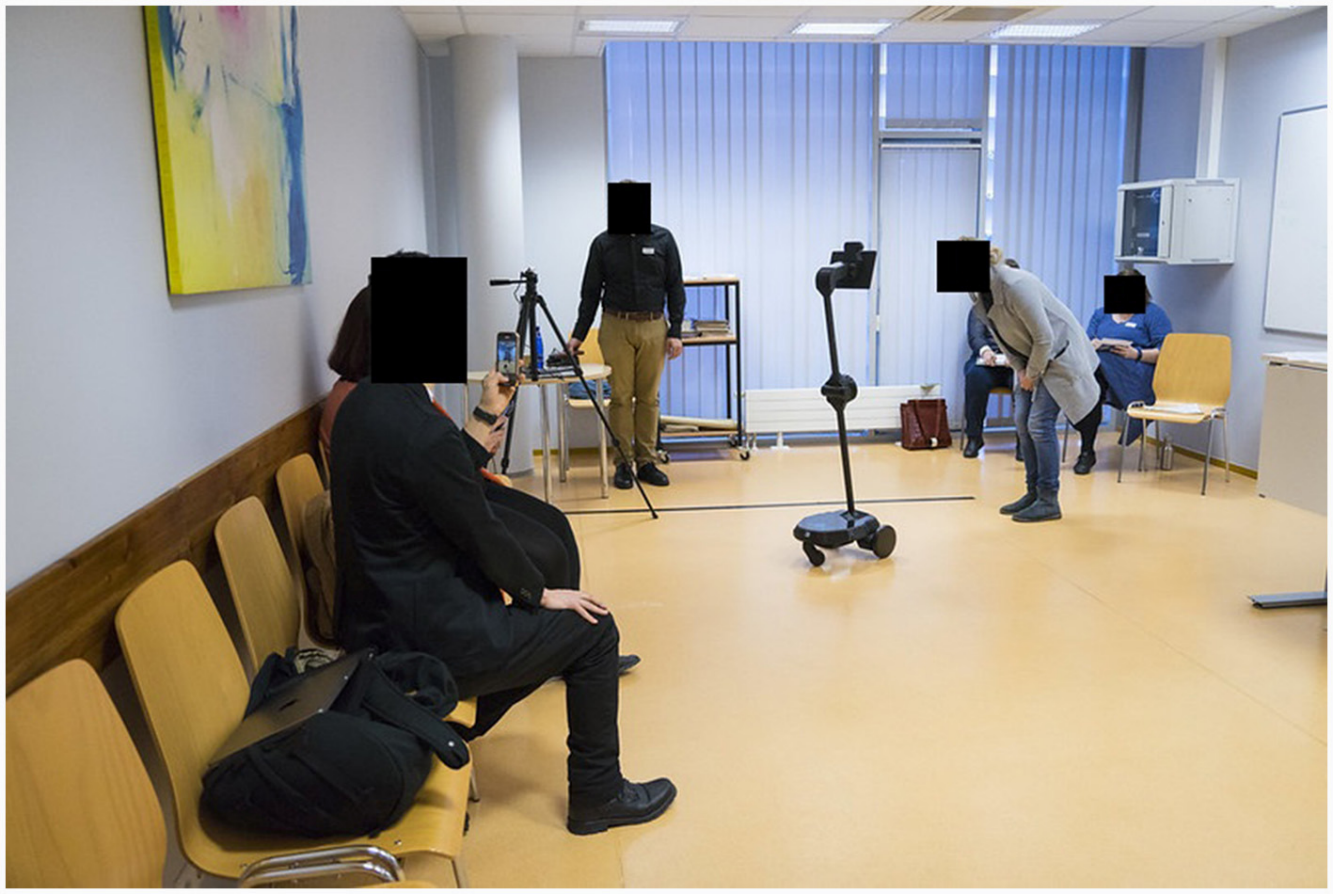
The layout of the communication room. The TP is interacting with a PP via a TPR **(center right)**. The other two PPs are waiting for their turn **(left)**. A technical person is standing near the camera. The supervisor is sitting **(back right)**.

The following persons were present in the communication room at the beginning of the communication situation: (a) three PPs; (b) three TPR mediated TPs (i.e., physically there were three TPRs in the room); (c) three observers, who each observed a different TPR and noted down their observations; (d) two technical persons who monitored the discussion situation sessions with a video camera and with a depth camera; and (e) a supervisor who introduced the experiment to participants and monitored the conduction of the experiment. The communication room layout is depicted on Figure 2.

**Figure 3:**
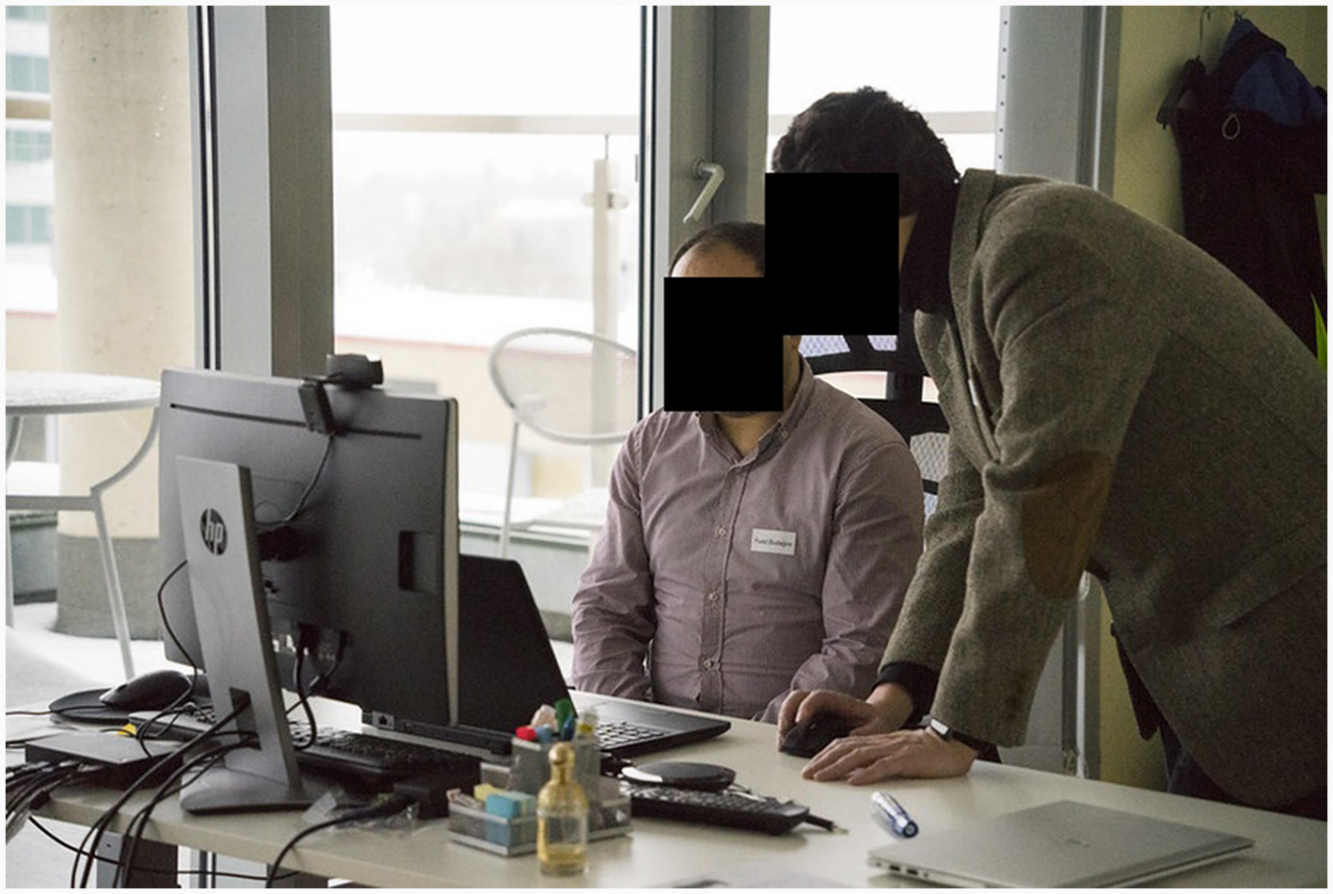
The layout of the control room. The TP is sitting behind the computer that is used to control the TPR. An observer-assistant (standing) is guiding the TP.

The following persons were present in each of the control rooms: (a) one TP (in-person); and (b) one observer-assistant, who instructed the TP for 3 min before the experiment, monitored the experiment and provided the TP with technical support when needed (Figure 3).

Instruction and training of the observers took place 2 weeks before the experiment and directly before the experiment on the spot. The observers were not allowed to interfere and they had to adhere to ethical principles (described in Section 2.3) during the observation.

Each group (from total four) of six participants (three PPs and three TPs) repeated the same experiment nine times, which means that each PP had a discussion with three different TPR-mediated TPs and each TP could be in charge of three different TPRs. For this end, the TPs in the robot control rooms switched places with each other after each communication situation was played out. Playing out each communication situation took about 3 min and the whole session for each group took about an hour. After all nine discussion situations had been played out, all participants in the sessions filled in a questionnaire on paper. After completing the questionnaire, the group was replaced with a new one.

#### 2.4.2 Description of communication situation

The PPs were sitting down. The first PP stood up, went to a certain location in the room and stayed there, holding a printed menu with font size 12 pt. The first TP approached, using the Double 3 robot, and stood at a comfortable distance from the PP with the menu. A conversation started with greetings, followed by a discussion about which restaurant to go to. The TP said that they knew a good restaurant and shared a photo of one of the dishes being offered. The PP looked at it. Then, the PP showed the menu to the TP and asked if it was the same menu. The TP had to read the menu aloud then. The communication situation ended, and the TP moved the TPR back. The scenario took about 3 min. Then, the next TP started to move the Ohmni robot, and the situation was repeated. The same was repeated with the TEMI 2 robot. To avoid repetitions, each robot operator had a menu of a different restaurant, and the people in the room had to choose the corresponding menu out of six menus.

After the first PP had played out the discussion situation with all three TPRs, the PP sat down, allowing the second PP to repeat the procedure. For the second round, all TPs switched the robots they were controlling. The third round with the third PP was conducted similarly, with all TPs switching their robots again. The impact of the familiarity of discussion participants on distance, arising from potential repetition, was mitigated by ensuring that there were no discussion situations with identical combinations of participants. We could not achieve full randomization of the discussion situations because our goal was to include groups with as much diversity in terms of gender and ethnicity as possible.

### 2.5 Data collection and analysis

The data about distance between the in-person and telepresent participants were collected using an Intel RealSense D415i depth-camera, which allows measuring distance between PPs and TPRs, and a regular video camera for control. A depth-camera records a distance from a camera for each pixel in a video. A single depth-camera frame from a greetings phase at the beginning of the conversation was chosen from a video stream for each experiment. The frame was chosen at a time step where both of the parties (the PP and TP) had chosen their positions and started a conversation. The frames were saved and (using markers) analyzed for distance between the communication parties. The markers were placed at the point closest to the PP on the TPR's upper body, and at the point closest to the TPR at the PP's face. The distance between those two points was calculated and recorded into a spreadsheet.

The observers had to note down information regarding problems, failures, abnormalities, etc. that could have had an impact on results. The observations were marked on a separate A4 sheet for each observation, with space for notes allocated for each unit of meaning. The observation protocols were digitized after the experiment and joined with the data extracted from the camera recordings.

The participants were asked to fill in a survey on paper right after the experiment. The surveys were digitized later and analyzed with the Excel spreadsheet software and the SPSS statistics software.

**Figure 4:**
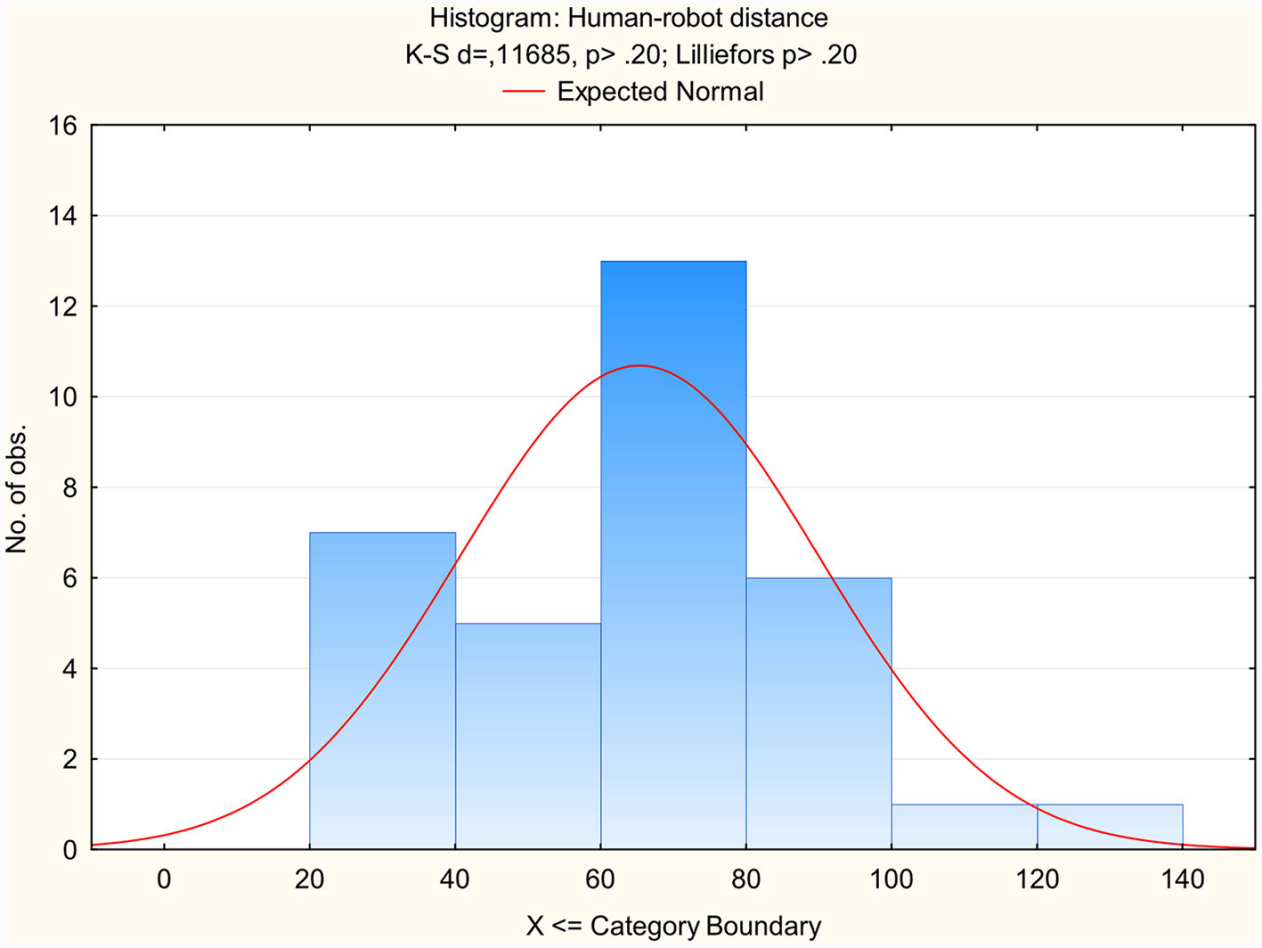
Normality testing results for distance between a TPR and a physically present person, as chosen by telepresent persons.

The normality of the data was confirmed (see Figure 4), taking into account that normality testing with relatively small sample sizes (< 50) can have certain complications: deviations from normality typically do not significantly impede hypothesis testing; normality tests exhibit limited power to reject the null hypothesis for small samples, resulting in their frequent acceptance for such cases (Ghasemi and Zahediasl, 2012; Knief and Forstmeier, 2021).

## 3 Results

### 3.1 Communication distance chosen by TP

Our first research question was “*What is the communication distance chosen by a TP when communicating with a PP, and does it differ from the communication distance chosen by a PP when communicating with a TP?*”

**Figure 5:**
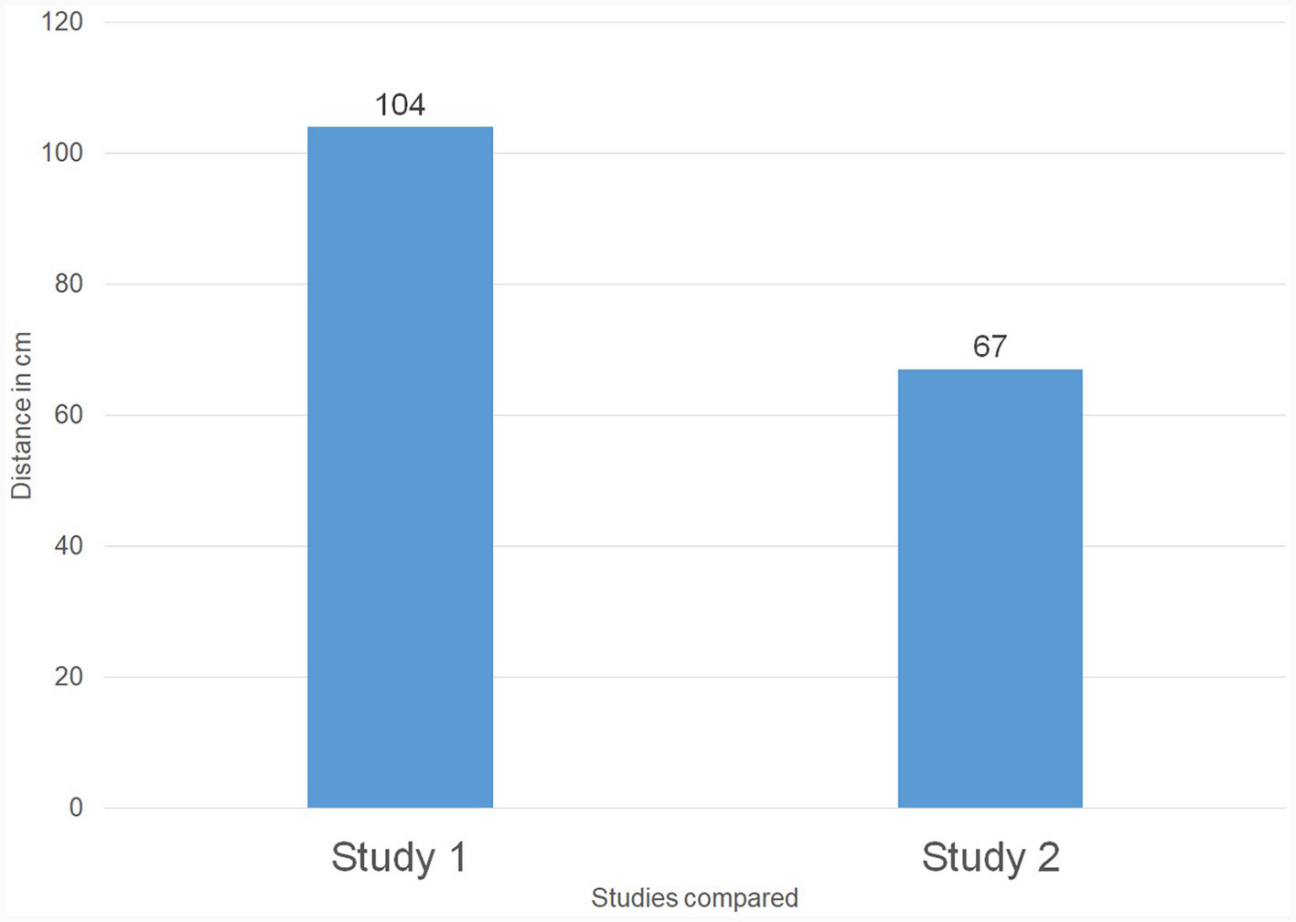
The average communication distance (in cm) chosen by physically present persons in Study 1 **(left)** compared to the one chosen by telepresent persons in Study 2 **(right)**.

**Figure 6:**
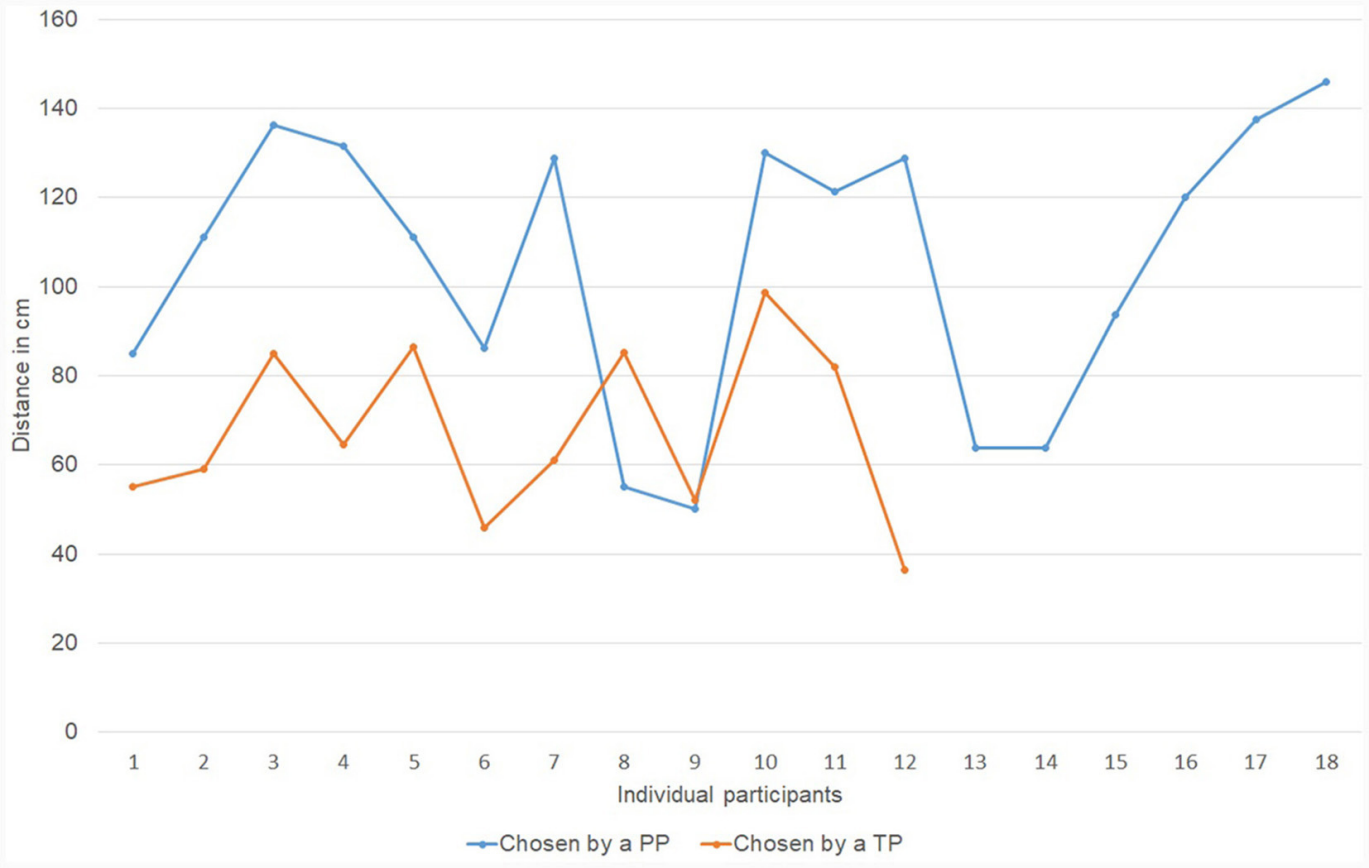
The communication distance (in cm) chosen by physically present persons in Study 1 (blue) compared to the one chosen by telepresent persons in Study 2 (orange). Each dot represents the average distance selected by an individual participant throughout valid iterations.

To answer this research question we compared the average communication distance (*M* = 104 cm; SD = 33.1 cm) that was chosen by PPs (*N* = 63) as measured in Study 1 (Leoste et al., 2023) with the average communication distance (*M* = 67 cm; SD = 23.8 cm) chosen by TPs (*N* = 33) as measured in Study 2 (this study), as demonstrated on Figures 4, 5 and 6. According to the *t*-test, the distances were statistically different: *t*_(32)_ = 6.25, *p* < 0.01.

### 3.2 Influence of TP's gender or the type of TPR used

Our second research question was “*Does the communication distance chosen by a TP depend on the TP's gender, or the type of TPR used?*”

**Figure 7:**
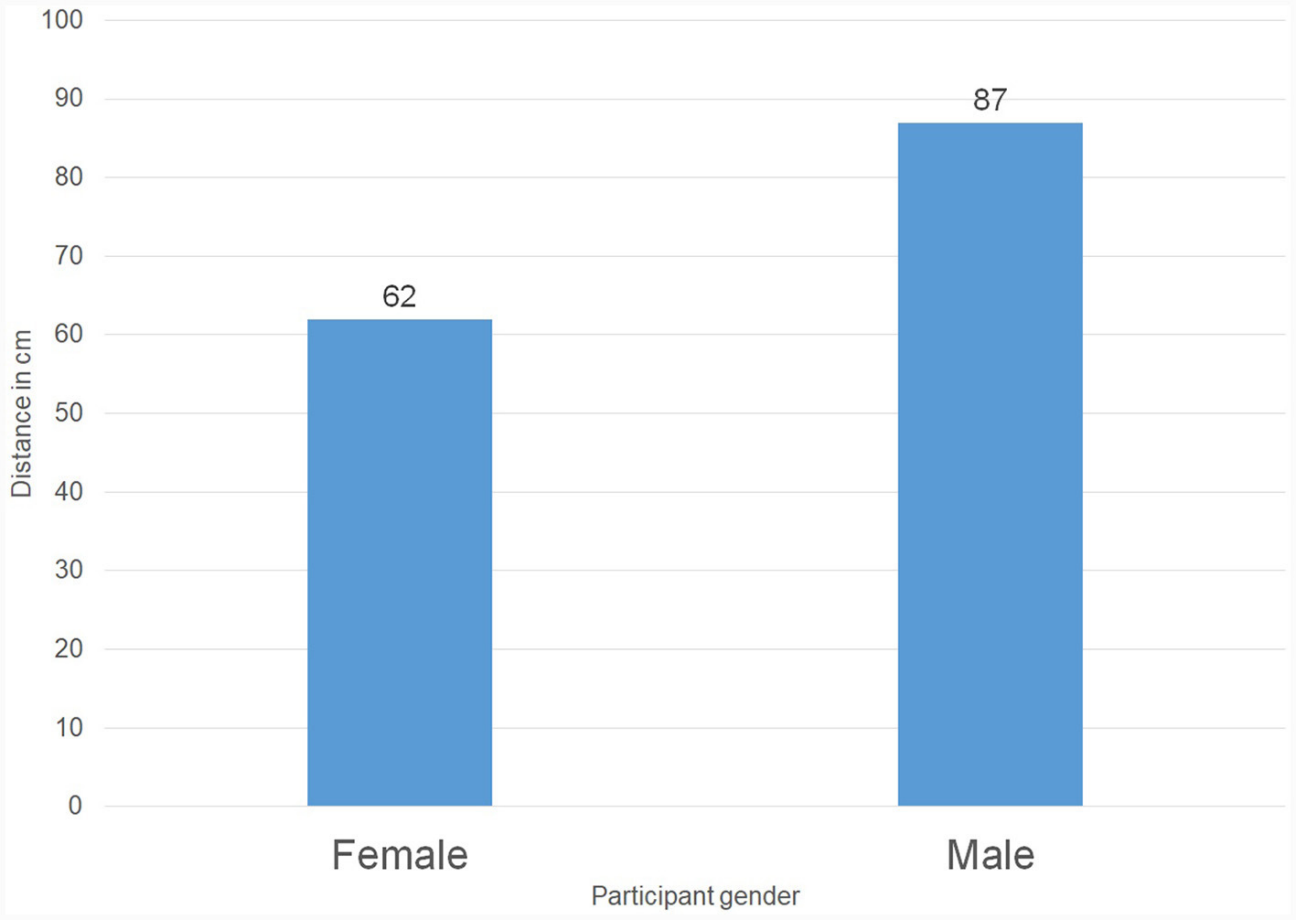
The average communication distance (in cm) chosen by female telepresent persons vs. male telepresent persons.

**Figure 8:**
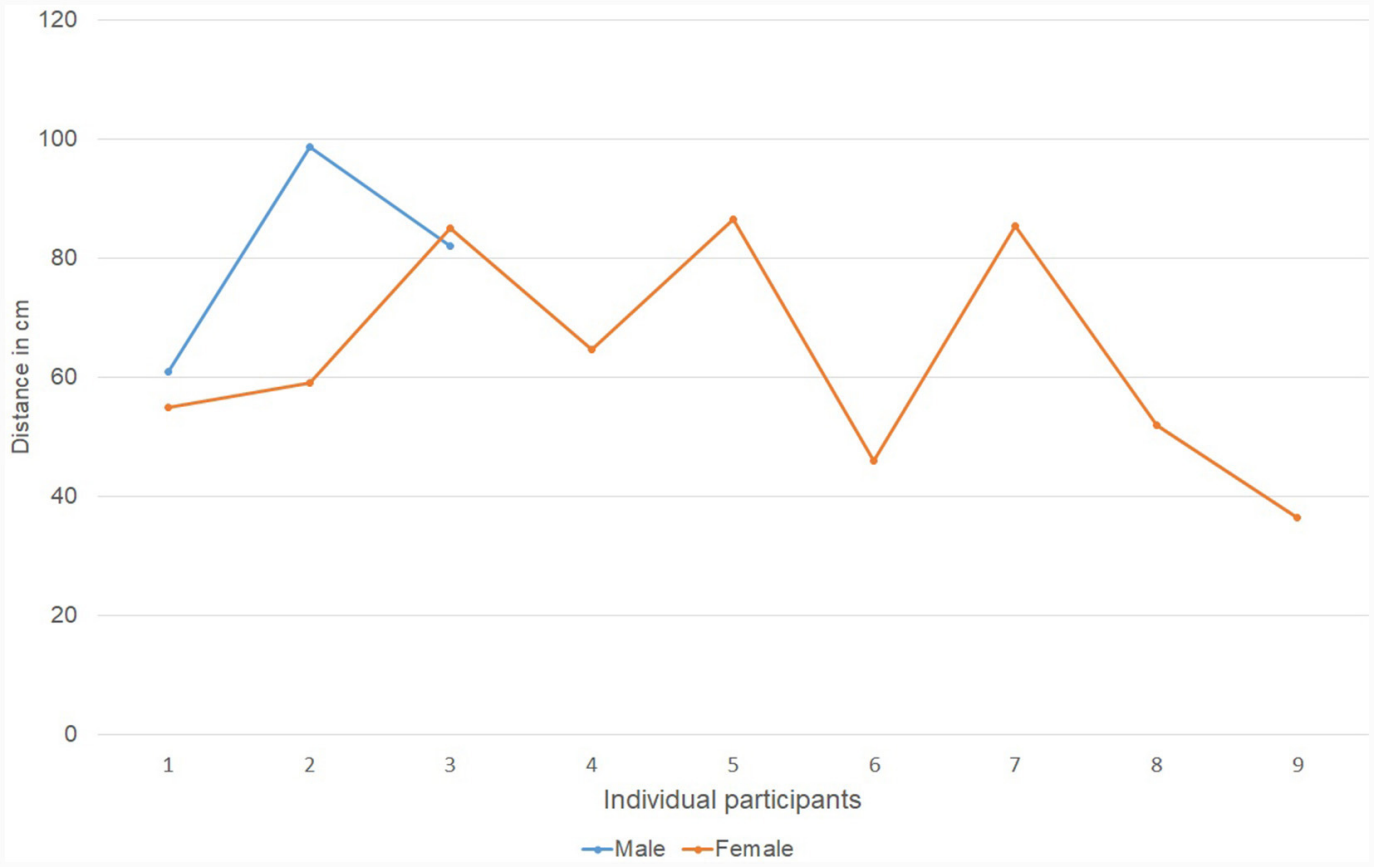
The communication distance (in cm) chosen by female telepresent persons vs. male telepresent persons. Each dot represents the average distance selected by an individual participant throughout valid iterations.

To answer this research question, we first compared the communication distances chosen by male TPs (*M* = 87 cm) to those of chosen by female TPs (*M* = 62 cm). The *t*-test indicates that these distances are statistically different: *t*_(32)_ = −2.67, *p* < 0.01. Male TPs chose a significantly larger communication distance (Figures 7, 8).

### 3.3 Behavioral realism of chosen communication distances

Our third research question was “*To what extent does the communication distance chosen by a TP differ from the ‘behaviorally realistic' distance that people maintain when communicating without a robot?*”

In order to determine the behaviorally realistic communication distance we departed from Hall's (1966) classification of communication distances. In our Study 1, about 80% of participants kept the communication distance of 60–160 cm, which, according to Hall, covers the far phase of personal distance and the close phase of social distance, and therefore may be considered as “behaviorally realistic.” The communication distances chosen by TPs in this study were significantly shorter (*M* = 67 cm), representing Hall's personal distance. As in Hall's theory, the personal communication distance is kept mainly with good acquaintances, and as the TPs in this study were not close acquaintances at all with the participating PPs, then this choice of communication distance cannot be considered as behaviorally realistic.

### 3.4 Technical suitability and subjective comfort of using TPR

Our fourth research question was “*How do TPs and PPs evaluate the technical suitability and subjective comfort of the TPR-mediated communication?*”

To understand the technical suitability and subjective comfort of the TPR-mediated communication we used the questionnaire that was piloted in Study 1 (Leoste et al., 2023). The questionnaire was filled in by the participating PPs and TPs immediately after the end of the experimental communication. In order to evaluate the technical suitability of the TPR-mediated communication the following statements were presented:

- *How successful do you think the communication was (were you able to convey your message to the people in the room)?* (Scale: 1-very poorly; 2-poorly; 3-not sure; 4-well; 5-very well).
- *How well could you hear your communication partners?* (Scale: 1-very poorly; 2-poorly; 3-not sure; 4-well; 5-very well).
- *How well did the robot obey your commands?* (Scale: 1-very poorly; 2-poorly; 3-not sure; 4-well; 5-very well).
- *How comfortable was it for you to read the text printed on paper shown by your communication partner?* (Scale: 1-very uncomfortable; 2-uncomfortable; 3-not sure; 4-comfortable; 5-very comfortable).

After being added up, the internal reliability of these statements was sufficient (α = 0.75) in order to use them as a scale for “technical suitability.” The scale's psychometric indicators were: *M* = 3.89; Min = 1.75; Max = 5.0; SD = 0.78.

To assess the subjective comfort of the TPR-mediated communication, the following two statements (or their one-statement alternative) were used:

- *How pleasant is this communication situation for you?* (1-very unpleasant; 2-unpleasant; 3-not sure; 4-pleasant; 5-very pleasant).
- *How comfortable did you find using a telepresence robot for communication, while you were in the robot and other people were physically present in the room?* (1-very uncomfortable; 2-uncomfortable; 3-not sure; 4-comfortable; 5-very comfortable).

or

- *How comfortable did you find using a telepresence robot for communication, while you were physically present in the room and the other person was in the robot?* (1-very uncomfortable; 2-uncomfortable; 3-not sure; 4-comfortable; 5-very comfortable).

After being added up, the internal reliability of these statements was sufficient (α = 0.77) in order to sum them up and use them as an indicator for “subjective comfort.” The scale's psychometric indicators were: *M* = 3.73; Min = 2.50; Max = 5.0; SD = 0.71.

As the mean value of both 5-point scales was almost 4, it allows to deduct that the majority of respondents considered the TPR-mediated communication as technically suitable and subjectively comfortable. From 33 respondents, six persons assessed communication comfort as low (mean value under 2.5), and three persons assessed the whole procedure as technically unsuitable.

These two assessments were not influenced by the responder's gender or their role as a TP or PP. Although the mutual correlation between these two scales was statistically significant (*r* = 0.28, *p* < 0.05), suggesting that the ability to cope with the TPRs' technical side could also affect the general pleasantness of the TPR-mediated communication.

### 3.5 Influence of the robot's type on technical suitability and subjective comfort

Our fifth research question was “*Do the assessments of TPs and PPs regarding the technical suitability and subjective comfort of the communication process depend on the type of robot used?*”

The differences between the three used types of TPRs were evaluated with a one-way ANOVA. As for subjective comfort, there were no differences between the robots. However, based on technical suitability, the TPRs did differ significantly. A one-way ANOVA revealed a statistically significant difference between the three types of TPRs. The Double robot got the highest score (*M* = 4.24; SD = 0.64), followed by Ohmni (*M* = 3.88; SD = 0.95), and TEMI 2 (*M* = 3.54; SD = 0.58); *F*_(2.68)_ = 5.34, *p* = 0.007.

## 4 Discussion and conclusions

The two studies we examined indicate that the choice of distance differs for people who communicate as a telepresent (TP) person compared to a situation where they participated as a physically present (PP) person. In the former case, a “behaviorally realistic” distance is preferred, while in the latter, a significantly closer/smaller distance is chosen. PPs choose more “normal” distance (according to the Hall categories), when facing TPRs (Leoste et al., 2023), while the people who interact via TPRs, tend to get unreasonably close/intimate. Which in turn means that a TP person can put the other communication partner in an uncomfortable situation.

At least some of the reasons for such “abnormal” distance choice may be technical in nature. When communicating as a TP, people lack distance experience, and excessive proximity could be due to a natural desire to see the conversation partner's face on the screen as large and detailed as in ordinary face-to-face communication, to see the partner's facial expressions, make eye contact, etc. The different field of view offered through a TPR may also have an effect, which in turn depends on the type of robot used.

The resulting message could be that when introducing TPRs, for example in education, the situations with greater intrapersonal distances (e.g., a person is present in the lecture hall via telepresence) require significantly more practice/training compared to situations where a PP communicates with a TP person in the audience. There is reason to believe that in the latter situation the robot (and the person behind it) is perceived as “behaviorally realistic” much faster than in vice versa communication situations.

In our sample, the choice of distance did not depend on robot type. However, it does depend on person's gender, and the results are somehow contradictory: women move closer when they “are in the robot,” while men do so when communicating “with the robot”. When acting as TPs (in TPRs), women move significantly closer to their communication partner (*M* = 62 cm) than men (*M* = 87 cm). However, as it was revealed in Study 1, when communicating as PPs, the opposite is true: men choose shorter distances (*M* = 87 cm) than women (*M* = 115 cm). There may be several reasons for this result, besides being connected to this specific and small sample. For example, female participants could dislike TPRs' robotic bodies more than men (Mumm and Mutlu, 2011); male participants could provide more personal space for persons that are perceived as disabled (see Kilbury et al., 1996); but the different choice of distance can be also connected to differently perceived social presence of the other communication party (Garau, 2003; Bailenson et al., 2005). Finally, the different choice of distance could be caused by the technical characteristics of TPRs, leading to distortions when mediating the real-world environment to TPs—the different focal length of TPRs' cameras combined with its small height could make it difficult for TPs to estimate distances correctly.

As for the technical suitability and subjective comfort, the TPRs in our study received mostly positive feedback from the participants. This positivity could be a result of the so-called novelty effect—as most of the participants were not routine users of TPRs, they might have perceived robots as novel and exciting. At the same time, the three types of TPRs used in the study were evaluated quite differently. Different factors may have a play here, from the usability of computer interface when controlling a TPR, to the TPR's height, the clarity of its display or resolution of its camera feed. These factors should be investigated in a dedicated separate study.

Our study is a piloting one and has several limitations. First, the sample in future studies should be larger and include wider diversity in order to represent groups with different social and cultural backgrounds more evenly. Second, the scale we used in this and in our previous study (Study 2 and Study 1) is still in a stage of piloting. For future studies, the scale should be more tested and maybe supplemented with question blocks about the TPR's features. Subsequently, scripted communication scenarios were employed, and the data collection mechanisms were readily identifiable. The studies ought to be replicated in authentic environments, employing less obtrusive methods for data collection. Lastly, forthcoming research endeavors should comprehensively investigate non-verbal language cues. In human communication, the significance of gaze and leaning is evident right from the initial contact moment, a fact validated also in human-robot interaction by Kanda et al. (2008) and Jirak et al. (2022). In these situations, of course, the researchers need to acknowledge the technical limitations of TPRs, such as relatively rudimental body or limited eye-sight, as highlighted by Talisainen et al. (2023).

In future research the communication distances under specific situations and environments (classrooms, hospitals, nursery homes, etc.) should be studied as well as combined with other aspects of non-verbal behavior—orientation, gestures etc. For example, do people keep shorter distances with TPs who are their close acquaintances, or would they keep larger distances with people of higher social status? Furthermore, we plan to enhance the depth of our research by broadening the study's focus to include individuals beyond direct communication scenarios. A more intricate analysis of the non-verbal parameters of communication through TPRs is imperative, encompassing the examination of non-verbal patterns exhibited by individuals in the immediate vicinity of the communication setting. Next, the impact of gender or different cultural background should be examined. Future studies should offer understanding or hints for questions, such as—how do we react when someone watches a play in the theater via a TPR? Or—how would people react if we would walk in a park with a TPR-mediated companion? These and other future studies should aim at supporting the development of TPRs toward “behavioral realism”, but also suggest how to teach people to use TPRs more efficiently.

## Data availability statement

The datasets presented in this study can be found in online repositories. The names of the repository/repositories and accession number(s) can be found below: https://drive.google.com/drive/folders/1erMkR9k8YabNxb4k9Qewea_3hF6yGteY?usp=sharing.

## Ethics statement

Ethical review and approval was not required for the study on human participants in accordance with the local legislation and institutional requirements.

## Author contributions

KM: Conceptualization, Data curation, Formal analysis, Investigation, Methodology, Visualization, Writing—original draft, Writing—review & editing. JL: Conceptualization, Data curation, Investigation, Methodology, Project administration, Supervision, Visualization, Writing—original draft, Writing—review & editing. MH: Conceptualization, Formal analysis, Methodology, Validation, Writing—review & editing. KK: Investigation, Validation, Writing—original draft. MR: Investigation, Software, Writing—original draft. JP: Investigation, Methodology, Writing—original draft. TK: Investigation, Writing—original draft.
